# Supplementary material for: Comparison of traditional systemic analgesic, single shot or continuous fascia iliaca compartment block for pain management in patients with hip or proximal femoral fractures: A protocol for systematic review and network meta-analysis
Source: PLoS One. 2025 Mar 26;20(3):e0319988. doi: 10.1371/journal.pone.0319988 (PMC11940658; doi:10.1371/journal.pone.0319988)
Supplement: S2 Table — (DOCX) [file pone.0319988.s002.docx]

| **S2 Table. The search strategy for PubMed.** | |
| --- | --- |
| **No.** | **Search items** |
| 1 | "Hip Fractures"[Mesh] |
| 2 | "Femoral Fractures"[Mesh] NOT "Femoral Fractures, Distal"[Mesh] |
| 3 | ((((((((((((((((((((((((((((Femoral Fracture*[Title/Abstract]) OR (Femoral Fracture, Proximal[Title/Abstract])) OR (Femoral Fractures, Proximal[Title/Abstract])) OR (Femoral Trochlear Fracture*[Title/Abstract])) OR (Femur Fracture, Proximal[Title/Abstract])) OR (Femur Fractures, Proximal[Title/Abstract])) OR (Femur Trochlear Fracture*[Title/Abstract])) OR (Fracture, Femoral[Title/Abstract])) OR (Fracture, Femoral Trochlear[Title/Abstract])) OR (Fracture, Femur Trochlear[Title/Abstract])) OR (Fracture, Proximal Femoral[Title/Abstract])) OR (Fracture, Proximal Femur[Title/Abstract])) OR (Fractures, Femoral[Title/Abstract])) OR (Fractures, Femoral Trochlear[Title/Abstract])) OR (Fractures, Femur Trochlear[Title/Abstract])) OR (Fractures, Hip[Title/Abstract])) OR (Fractures, Intertrochanteric[Title/Abstract])) OR (Fractures, Proximal Femoral[Title/Abstract])) OR (Fractures, Subtrochanteric[Title/Abstract])) OR (Fractures, Trochanteric[Title/Abstract])) OR (Intertrochanteric Fracture*[Title/Abstract])) OR (Proximal Femoral Fracture*[Title/Abstract])) OR (Proximal Femur Fracture*[Title/Abstract])) OR (Subtrochanteric Fracture*[Title/Abstract])) OR (Trochanteric Fracture*[Title/Abstract])) OR (Trochlear Fracture, Femoral[Title/Abstract])) OR (Trochlear Fracture, Femur[Title/Abstract])) OR (Trochlear Fractures, Femoral[Title/Abstract])) OR (Trochlear Fractures, Femur[Title/Abstract]) |
| 4 | #1 OR #2 OR #3 |
| 5 | (((((fascia iliaca block[Title/Abstract]) OR (Fascia iliaca compartment block[Title/Abstract])) OR (Iliofascial block[Title/Abstract])) OR (Iliac Fascial Block[Title/Abstract])) OR (FIB[Title/Abstract])) OR (FICB[Title/Abstract]) |
| 6 | "Analgesia, Patient-Controlled"[Mesh] |
| 7 | (((((((((((Analgesia, Patient Controlled[Title/Abstract]) OR (Patient-Controlled Analgesia[Title/Abstract])) OR (Patient Controlled Analgesia[Title/Abstract])) OR (patient controlled intravenous analgesia[Title/Abstract])) OR (PCIA[Title/Abstract])) OR (opioid*[Title/Abstract])) OR (morphine[Title/Abstract])) OR (fentanyl[Title/Abstract])) OR (sufentanil[Title/Abstract])) OR (conventional analgesia[Title/Abstract])) OR (systemic analgesia[Title/Abstract])) OR (intravenous analgesic[Title/Abstract]) |
| 8 | #5 OR #6 OR #7 |
| 9 | (randomized controlled trial[Publication Type]) OR (random*[Title/Abstract]) |
| 10 | #4 AND #8 AND #9 |
