# Supplementary material for: Comparison of traditional systemic analgesic, single shot or continuous fascia iliaca compartment block for pain management in patients with hip or proximal femoral fractures: A protocol for systematic review and network meta-analysis
Source: PLoS One. 2025 Mar 26;20(3):e0319988. doi: 10.1371/journal.pone.0319988 (PMC11940658; doi:10.1371/journal.pone.0319988)
Supplement: Attachment — Submitted filename: 1_Comment for Traditional systemic analgesic_protocol for review.doc [file pone.0319988.s003.doc]

**Comments**

**General comments**

As there are a number of omissions in the design of this study and written English is not academic standard, the value of this topic under review is questionable.

**Abstract/ Introduction-content**

No read

**Methods: General**

-It would be better to have two subtitles as “Identification and selection of studies” and “Assessment of characteristics of studies”.

-Information is messy, so it makes readers feel difficult to read.

**Methods: Identification and selection of studies**

Information is messy. Inclusion criteria should be following as Design (missing what kind of design would be included), Participants (not clear), Outcome measures (no enough information)

***Searches***

1. For each database, it should be as example like: Embase (1947 to June 2023). This is missing

***Inclusion criteria: this should be part of Identification and selection of studies***

Authors should tidy up information, perhaps using a table to cover all area, such as Design, Participants, Intervention, Outcome measures, Comparisons.

**Methods: Assessment of characteristics of studies**

There should be more points in this part. It would be better to have this headings and follow some subheading as below:

*Quality:* missing

*Participants:* missing

*Intervention:* messing

*Outcome measures:* missing

**Results/ Discussion/ Conclusion: context.**

As there are so many questions in the method, results, discussion and conclusion are questionable.

**Readability and style.**

This paper is not easy to read, because the structure of written information.
