# Supplementary material for: Comparison of traditional systemic analgesic, single shot or continuous fascia iliaca compartment block for pain management in patients with hip or proximal femoral fractures: A protocol for systematic review and network meta-analysis
Source: PLoS One. 2025 Mar 26;20(3):e0319988. doi: 10.1371/journal.pone.0319988 (PMC11940658; doi:10.1371/journal.pone.0319988)
Supplement: Attachment — Submitted filename: Response_to_Reviewers_auresp_2.docx [file pone.0319988.s006.docx]

Jiaxi Tang MD. PhD

**Department of Anesthesiology, Chongqing Key Laboratory of Translational Research for Cancer Metastasis and Individualized Treatment, Chongqing University Cancer Hospital,**

Chongqing 400030, China

E-mail: tangjiaxi1029@126.com

December 3, 2024

**RE:  Manuscript ID:** PONE-D-23-33546R1

Dear Editor，

We would like to thank the editor for giving us a chance to resubmit the paper, and also thank the reviewer for giving us constructive suggestions which would help us in depth to improve the quality of the paper. Here we submit a new version of our manuscript with the title “**Comparison of traditional systemic analgesic, Single shot or continuous Fascia Iliaca Compartment Block for pain management in patients with hip or Proximal Femoral Fractures: A protocol for systematic review and network meta-analysis**”, which has been modified according to the reviewer and editor’s suggestions. We highlight all the changes using a track change function in the revised manuscript.

Sincerely yours,

Jiaxi Tang MD. PhD

-------------------------------------------------------------------------------------------------------

The following is a point-by-point response to the editor and reviewer’s comments.

**Editor:**

**General comments:**

Although your corrections to the previous reviews have improved the manuscript, it still needs attention to several details and clarifications.

**Answer:** Thank you for comments and guidance on the paper, and thanks for giving us the opportunity to revise it. We have revised our manuscript based on comments from editor and reviewer to make it more readable and clear.

**Specific Comments:**

1. In Introduction:
1) Provide a brief background on the systemic analgesics and fascia iliaca nerve blocks as how they provide analgesia for hip or proximal femoral fractures.

**Answer:** Thank you for your valuable advice. Our previous manuscript did not provide a brief background on the systemic analgesic regimen and fascia iliaca nerve blocks for hip or proximal femoral fractures analgesia, which may be unfriendly to non-specialists and add to the difficulty and confusion of reading. We have added this section, thanks again. (Line 60-69)

2) Comment on what type of pain and analgesia duration the investigators are attempting to address.

**Answer:** Thank you for your insightful comment regarding the type of pain and analgesia duration addressed in our study. We included patients with hip fractures regardless of whether they underwent surgery to reflect the diversity of real-world clinical scenarios. Our study did not specifically focus on preoperative, postoperative, or admission-related pain as our primary goal was to evaluate the overall efficacy of analgesic modalities in managing acute pain during hospitalization. In addition, we have included a specific time frame for pain assessment in the methodological section to enhance the clarity of our research.(Line 85-88, 184-186)

To address this complexity, our study protocol includes a predefined subgroup analysis based on the timing of nerve block administration. This analysis will allow us to explore the impact of nerve block timing on analgesic outcomes and provide more nuanced insights into pain management strategies across different stages of care.(Line 240-252)

3) It’s a well-known phenomenon that pain management for joint surgery requires multimodal analgesia. So, what is the rationale behind comparing systemic analgesia vs peripheral nerve blocks?

**Answer:** Thank you for highlighting the importance of multimodal analgesia in joint surgery pain management. We agree that such approaches are essential. The rationale for our comparison between systemic analgesia and peripheral nerve blocks is to explore their individual roles within multimodal strategies. Systemic analgesics, though effective and widely accessible, can lead to dose-dependent side effects. Peripheral nerve blocks, on the other hand, offer localized pain relief with fewer systemic effects but require specialized resources.

By comparing these methods, our study aims to provide evidence that helps clinicians optimize multimodal analgesia, balancing efficacy, safety, and resource considerations to meet the needs of diverse patient populations.(Line 85-88)

4) What is the rationale behind investigating a single shot vs a continuous nerve block?

**Answer:** Thank you for raising this important point. Single-shot nerve blocks provide effective pain relief; however, their duration is inherently limited, often necessitating supplemental analgesia, such as systemic opioids, to manage breakthrough pain. Additionally, continuous FICB requires specialized equipment and techniques and carries risks such as weakened quadriceps strength, delayed recovery, and prolonged hospital stays. These limitations are the key reasons for investigating the comparative efficacy of single-shot versus continuous nerve blocks. Our study aims to evaluate how these approaches address acute pain in hip fracture patients and inform optimal analgesic strategies that balance efficacy, safety, and resource utilization. (Line 81-83)

2. In materials and methods:

1) Comment on how studies including shaft and distal femoral fractures will be reliably excluded during the search.

**Answer:** Thank you for your question regarding the exclusion of shaft and distal femoral fractures during the search. To ensure reliable exclusion, we designed our search strategy to explicitly differentiate between proximal femoral fractures and other types of femoral fractures. Specifically, as shown in Supplementary Table S2, we used the MeSH term "Femoral Fractures"[Mesh] and excluded "Femoral Fractures, Distal"[Mesh] with the NOT operator.(S2 Table) We also made changes in the manuscript. (Line 110-111)

2) Inter variability between the types of fascia iliaca blocks and the providers who perform them have a significant influence on outcomes, hence will generically including all under one umbrella be a meaningful investigation?

**Answer:** Thank you for highlighting the potential inter-variability between different types of fascia iliaca blocks (FIB) and the providers who perform them. We fully agree that these factors can significantly influence analgesic outcomes and warrant careful consideration.

To address this, our study protocol includes predefined subgroup analyses that will stratify outcomes based on the specific FIB techniques (e.g., suprainguinal vs infrainguinal approaches), methods (e.g., landmark-based vs ultrasound-guided), and the provider performing the block (e.g., anesthesiologists vs emergency department physicians). By incorporating these subgroup analyses, we aim to better understand the impact of these variables on the efficacy of FIB and to provide more nuanced insights for clinical practice.

We believe this approach balances the need for capturing real-world diversity with the importance of accounting for key sources of variability, ensuring the robustness and applicability of our findings. (Line 176-181, 240-252)

3) Define “abstracts with sufficient data”.

**Answer:** Thank you for pointing out the need to define "abstracts with sufficient data." In our study, this term refers to abstracts that provide adequate information to assess eligibility and extract key outcomes, such as patient population, intervention (fascia iliaca nerve block), comparator (systemic analgesia), and primary or secondary outcomes related to analgesic efficacy or safety.

This definition ensures that only abstracts with the necessary details to contribute to our analysis are included, while abstracts lacking critical information (e.g., no description of the intervention or outcomes) are excluded. We have now included this definition in the manuscript for clarity. (Line 125-129)

4) Shouldn’t the fascia iiaca block studies either be inclusive or exclusive of systemic analgesia as they will eventually impact the final outcomes.

**Answer:** Thank you for your thoughtful comment regarding the inclusion or exclusion of systemic analgesia in fascia iliaca block (FICB) studies. We agree that systemic analgesia can have a significant impact on the final outcomes, and it is important to consider how its use may influence the effectiveness of FICB.

In our study, we have decided to include studies regardless of whether systemic analgesia was used in combination with FICB. This approach reflects real-world clinical practice, where FICB is often used alongside systemic analgesia to provide more comprehensive pain management.

To compare multiple analgesic treatments in this study, we plan to conduct a network meta-analysis and plot cumulative ranking curves (SUCRA) based on key outcomes and adverse events to assess the benefits and risks of various analgesic regimens. (Line 228-230)

5) Clarify the duration when the primary outcomes are going to be investigated.

**Answer:** Thank you for your comment regarding the duration for assessing the primary outcomes. The primary outcome in our study is pain score, which will be measured at predefined time points based on clinical relevance. Specifically, Pain will be assessed at predefined time points: before treatment, immediately after treatment, and at 6, 24, and 48 hours post-treatment, which are chosen to capture both the immediate and long-term effects of the analgesic treatments.

We believe that these time points will offer a comprehensive understanding of the analgesic efficacy and pain relief provided by different interventions for acute pain in hospitalized patients. (Line 183-188)

6) Clarify the outcome measure technical success. How will the successful injection of local anesthetic be determined?

**Answer:** Thank you for your comment. In this systematic review, we define technical success based on criteria commonly reported in the included studies. Specifically, technical success is confirmed by one or more of the following:

Ultrasound Confirmation: Successful visualization of the local anesthetic within the fascia iliaca compartment.

Loss of resistance: When the tip of the needle passes through the fascia lata and the fascia iliaca, a loss of resistance is felt, as commonly described in the literature.

Or other criteria specified by the authors (Line 193-195)

7) Define the secondary outcomes such as adverse events, complications and patient satisfaction

**Answer:** Thank you for your insighted suggestion. Defining secondary indicators in advance will make the evaluation of secondary indicators in this system evaluation more transparent and targeted. We have revised it according to your comments. (Line 189-203)

**Reviewer #3:**

**General comments:**

1. Does the manuscript provide a valid rationale for the proposed study, with clearly identified and justified research questions?

The research question outlined is expected to address a valid academic problem or topic and contribute to the base of knowledge in the field.

Reviewer #3: Yes

________________________________________

2. Is the protocol technically sound and planned in a manner that will lead to a meaningful outcome and allow testing the stated hypotheses?

The manuscript should describe the methods in sufficient detail to prevent undisclosed flexibility in the experimental procedure or analysis pipeline, including sufficient outcome-neutral conditions (e.g. necessary controls, absence of floor or ceiling effects) to test the proposed hypotheses and a statistical power analysis where applicable. As there may be aspects of the methodology and analysis which can only be refined once the work is undertaken, authors should outline potential assumptions and explicitly describe what aspects of the proposed analyses, if any, are exploratory.

Reviewer #3: Yes

________________________________________

3. Is the methodology feasible and described in sufficient detail to allow the work to be replicable?

Descriptions of methods and materials in the protocol should be reported in sufficient detail for another researcher to reproduce all experiments and analyses. The protocol should describe the appropriate controls, sample size calculations, and replication needed to ensure that the data are robust and reproducible.

Reviewer #3: Yes

________________________________________

4. Have the authors described where all data underlying the findings will be made available when the study is complete?

The PLOS Data policy requires authors to make all data underlying the findings described in their manuscript fully available without restriction, with rare exception, at the time of publication. The data should be provided as part of the manuscript or its supporting information, or deposited to a public repository. For example, in addition to summary statistics, the data points behind means, medians and variance measures should be available. If there are restrictions on publicly sharing data—e.g. participant privacy or use of data from a third party—those must be specified.

Reviewer #3: Yes

________________________________________

5. Is the manuscript presented in an intelligible fashion and written in standard English?

PLOS ONE does not copyedit accepted manuscripts, so the language in submitted articles must be clear, correct, and unambiguous. Any typographical or grammatical errors should be corrected at revision, so please note any specific errors here.

Reviewer #3: Yes

**Answer:** Thank you for your comments and recognition of our research.

**Specific Comments:**

1. Studies

Line 117-118 mentions the inclusion of all analgesia regimens, including fascia iliaca compartment block (single-injection or continuous infusion) and systemic analgesia alone. Could you clarify whether your review will include only studies comparing single-shot vs. continuous FICB, or if studies comparing FICB to placebo or systemic analgesia to placebo will also be included? If so, how will this align with your hypothesis that continuous FICB may prolong analgesic effects and reduce opioid use compared to single-shot or systemic analgesia?

**Answer:** Thank you for your insightful comments and for the opportunity to clarify our study design.
Regarding your query about the inclusion criteria for our review (Lines 117-118), we confirm that our study will include comparisons among the following analgesic regimens, as illustrated in Figure 2:
1. Single-shot FICB (± systemic analgesia)
2. Continuous FICB (± systemic analgesia)
3. Systemic analgesia alone (e.g., opioids or multimodal analgesia)

However, we will not include studies comparing systemic analgesia to placebo studies. In cases of hip fracture, the absence of analgesia is neither ethically permissible nor practically feasible; thus, such studies are unlikely to exist or be eligible for inclusion.

Furthermore, we acknowledge that studies directly comparing single-shot FICB to continuous FICB are limited. To address this, we plan to use a network meta-analysis (NMA) approach. This method enables indirect comparisons through a connected network of studies, incorporating data from multiple analgesic regimens. By leveraging this framework, we aim to comprehensively assess the relative efficacy and safety of single-shot versus continuous FICB, even in the absence of abundant direct comparative studies.

We appreciate your thoughtful feedback, which has helped us to clarify and strengthen the description of our study methodology. Please let us know if further clarification is needed. (Line 221-232, Figure2)

2. Data Collection

Could you discuss potential challenges in collecting data from the included studies, especially considering variations in how different analgesic techniques are administered (e.g., differences in dosage, technique, or patient populations)? This would help clarify the limitations of the meta-analysis.

**Answer:** Thank you for raising this critical point regarding potential challenges in data collection and the implications for our meta-analysis. Variations in how analgesic techniques are administered, including systemic analgesia regimens, indeed present challenges for synthesizing data.

We have revised the discussion section and the method section to address the limitations of the study and solutions to the problem.(Line 240-252, 265-285).

3. Outcome Measures

While you mention outcome measures, could you provide more details on how pain scores, mobility, and adverse events will be standardized across studies to manage potential heterogeneity?

**Answer:** Thank you for your constructive comments. This is essential to clarify the outcome measures criteria for our studies and to facilitate replication of our studies by other researchers. We have established the criteria for outcome measures. (Line 183-203).

4. Discussion

How do you envision translating the findings from this study into clinical practice, particularly with respect to updating pain management guidelines for hip fractures?

**Answer:** Thank you for your insightful question. The findings from our systematic review and network meta-analysis aim to identify the most effective and safe analgesic strategies for hip fracture pain management. By synthesizing high-quality evidence and ranking the comparative efficacy and safety of various analgesic regimens, we hope to provide actionable recommendations that can directly inform clinical practice.

Our results could support the development and revision of pain management guidelines by highlighting the benefits and limitations of single-shot versus continuous FICB, as well as systemic analgesia regimens. Additionally, these findings could assist clinicians in tailoring pain management strategies based on patient characteristics, improving outcomes and patient satisfaction. By collaborating with guideline committees and presenting our findings at relevant clinical forums, we anticipate translating these results into updated, evidence-based clinical guidelines for managing hip fracture pain. (Line 286-295)
