# Supplementary material for: Comparison of traditional systemic analgesic, single shot or continuous fascia iliaca compartment block for pain management in patients with hip or proximal femoral fractures: A protocol for systematic review and network meta-analysis
Source: PLoS One. 2025 Mar 26;20(3):e0319988. doi: 10.1371/journal.pone.0319988 (PMC11940658; doi:10.1371/journal.pone.0319988)
Supplement: Attachment — Submitted filename: Response to Reviewers.docx [file pone.0319988.s005.docx]

Jiaxi Tang MD. PhD

**Department of Anesthesiology, Chongqing Key Laboratory of Translational Research for Cancer Metastasis and Individualized Treatment, Chongqing University Cancer Hospital,**

Chongqing 400030, China

E-mail: tangjiaxi1029@126.com

June 26, 2024

**RE:  Manuscript ID:** PONE-D-23-33546

Dear Editor，

We would like to thank the editor for giving us a chance to resubmit the paper, and also thank the reviewer for giving us constructive suggestions which would help us both in English and in depth to improve the quality of the paper. Here we submit a new version of our manuscript with the title “**Comparison of traditional systemic analgesic, Single shot or continuous Fascia Iliaca Compartment Block for pain management in patients with hip or Proximal Femoral Fractures: A protocol for systematic review and network meta-analysis**”, which has been modified according to the reviewers’ suggestions. Efforts were also made to correct the mistakes and improve the English of the manuscript. We highlight all the changes using a track change function in the revised manuscript.

Sincerely yours,

Jiaxi Tang MD. PhD

-------------------------------------------------------------------------------------------------------

The following is a point-by-point response to the editor and reviewers’ comments.

**Editor:**

**General comments:**

As there are a number of omissions in the design of this study and written English is not academic standard, the value of this topic under review is questionable.

**Answer:** Thank you for comments and guidance on the paper, and thanks for giving us the opportunity to revise it. We have revised our manuscript based on comments from editors and reviewers to make it more readable and clear.

**Specific Comments:**

1. Abstract/ Introduction-content

No read

**Answer:** Thank you for your comments. We will make revisions based on comments from editor and reviewers. Please review it again after the revision is completed. Thank you. (Line 32-36)

2. Methods: General

-It would be better to have two subtitles as “Identification and selection of studies” and “Assessment of characteristics of studies”.

-Information is messy, so it makes readers feel difficult to read.

**Answer:** Thank you for your enlightening advice, which can improve the quality and readability of our manuscripts. We have readjusted the order of the methods section, adding two subtitles: "Identification and selection of studies" and "Assessment of characteristics of studies". (Line 90; Line 133).

3. Methods: Identification and selection of studies

Information is messy. Inclusion criteria should be following as Design (missing what kind of design would be included), Participants (not clear), Outcome measures (no enough information)

Searches

For each database, it should be as example like: Embase (1947 to June 2023). This is missing

Inclusion criteria: this should be part of Identification and selection of studies

Authors should tidy up information, perhaps using a table to cover all area, such as Design, Participants, Intervention, Outcome measures, Comparisons.

**Answer:** Thank you for your comments and suggestions. We have updated the order of inclusion criteria in our manuscript. In addition, we have added information for parts of the manuscript that are not explicitly described, and have increased the specific search intervals for each databases. (Line 93-97; line 114-123).

4. Methods: Assessment of characteristics of studies

There should be more points in this part. It would be better to have this headings and follow some subheading as below:

Quality: missing

Participants: missing

Intervention: messing

Outcome measures: missing

**Answer:** Thank you for your enlightening comments, which will make our manuscript more organized and legible. We have revised this part according to your comments. (Line 153-180).

5. Results/ Discussion/ Conclusion: context.

As there are so many questions in the method, results, discussion and conclusion are questionable.

**Answer:** Thank you for your reminder and commets. We have revised the discussion section accordingly. (Line 219-229).

6. Readability and style.

This paper is not easy to read, because the structure of written information.

**Answer:** Thank you for your comments. We have revised the structure of the manuscript in response to comments from editors and reviewers. (Line 90-217).

**Reviewer #1:**

**General comments:**

This systematic review protocol and Network meta-analysis evaluates traditional systemic analgesia interventions with continuous or single shot anesthetic block of the fascia iliaca in patients with fractures of the hip or proximal femur regarding pain and the use of opioids. The methodology follows PRISMA recommendations.

I suggest that some points be reviewed by the authors:

- Introduction – The authors describe that although several studies, including systematic reviews, demonstrate that Fascia Iliaca compartment block is effective and reduces the use of opioids in patients with fractures of the proximal femur, this is correct. “However, Dai et.al[20] and Smith et.al[21] indicated that FICB is not superior to placebo for patients undergoing hip surgeries”, these two studies refer to arthroscopy and primary hip arthroplasty, surgeries that do not are the subject of this review and therefore should not be used as a rationale for preparing this protocol.

- Study Selection –

linhas 112-114-

Language: “Considering that there are no translators proficient in other languages, only full-text articles published in English or Chinese”. I suggest that there is no language restriction, for example, in the study by Slade (2023) two studies published in French were included.

Line 119- “ Exclusion criteria: (i) replicated and low-quality studies” – I suggest describing the criteria adopted to define which low-quality studies will be excluded.

- Plos data Policy - According to the Plos data policy, authors are required to describe where all data underlying the findings of their manuscript will be made available (database or public repository), without restrictions.

**Answer:** Thank you for your comments and reminder. We have revised our manuscript accordingly. I would also like to thank you for your valuable comments on the paper, which have made a great contribution to improving the quality and accuracy of the paper.

**Specific Comments:**

1. Introduction – The authors describe that although several studies, including systematic reviews, demonstrate that Fascia Iliaca compartment block is effective and reduces the use of opioids in patients with fractures of the proximal femur, this is correct. “However, Dai et.al[20] and Smith et.al[21] indicated that FICB is not superior to placebo for patients undergoing hip surgeries”, these two studies refer to arthroscopy and primary hip arthroplasty, surgeries that do not are the subject of this review and therefore should not be used as a rationale for preparing this protocol.

**Answer:** Thank you for your comments on the paper, which are of great help in improving the rigor of our manuscript. We have substituted a suitable reference to elucidate the rationale underlying our research. (Line 63-69).

2. Study Selection –

linhas 112-114-

Language: “Considering that there are no translators proficient in other languages, only full-text articles published in English or Chinese”. I suggest that there is no language restriction, for example, in the study by Slade (2023) two studies published in French were included.

**Answer:** Thank you for the comments on the paper. We have removed the restrictions on the language of publication. Indeed, limiting the language of the included study is not conducive to a comprehensive analysis of our topic. Thank you again for your valuable advice. (Line 123).

3. Line 119- “ Exclusion criteria: (i) replicated and low-quality studies” – I suggest describing the criteria adopted to define which low-quality studies will be excluded.

**Answer:** Thank you for your constructive comments. This is essential to clarify the inclusion and exclusion criteria for our studies and to facilitate replication of our studies by other researchers. We have established the criteria for low-quality studies based on previous literature. (Line 129).

4. Plos data Policy - According to the Plos data policy, authors are required to describe where all data underlying the findings of their manuscript will be made available (database or public repository), without restrictions.

**Answer:** Thank you for your comments and kind reminder. We will provide a complete Data Availability Statement in the submission form. Thanks again.

**Reviewer #2:**

**General comments:**

The respected authors have presented a Pilot study to assess the pain management strategies following a hip fracture. The authors tend to investigate the impact of the single and continuous fascia Iliaca Compartment Block technique in comparison to the conventional systemic analgesics, employing a Bayesian Network meta-analysis method. The manuscript will address a major clinical problem.

However, the author should address the following issues to improve the quality of the manuscript.

Introduction:

Lines 47-48: The author states the possible prevalence projection of hip fracture; however, the author needs to add statistical evidence from the listed references to provide support for their statement.

Lines 48-50: the author cited the 2011 Association of Anaesthetists, the author should cite the recent guideline published in 2020.

Line 68-69: the author should be emphasizing the hypothesis and not the methodology in the introduction. The statement for the Bayesian NMA and indirect comparison is more suitable in the method section and is irrelevant to the introduction.

Line 52-54: the statement is unclear please rewrite the statement for more clarity.

Line 72-73: the author presents their hypothesis in the former sentence, however, in lines 72-73, the author should state that they will assess the duration of lower limb weakness as a secondary outcome, however, the written statement looks like the author is quite sure about the possible outcome and its risk regarding the weakness. This statement is not relevant in writing an introduction.

Methods:

Line 91-92: the author included only the Fascia iliaca block as an intervention in the search strategy. Similarly, table S1 also shows the strategy only restricted to the following keywords. In contrast, the network diagram shows systemic analgesics, similarly, the inclusion criteria also include systemic analgesics in lines 107-108. Therefore, systemic analgesia should also be included in the search strategy to provide the rank-based analysis of all the interventions considering the author is planning to conduct a network meta-analysis. However, according to the hypothesis, the author seems only interested in the single and continuous FICB so a meta-analysis or a network analysis only with the three interventions: single, continuous FICB and placebo should be conducted, along with an update of the illustrated network plot will be required. The author needs to refine and redefine their methodology, search strategy, PICO and hypothesis regarding the inclusion or exclusion of systemic analgesics.

Line 164: the statement is unclear.

Discussion

Line 181: the author claims the novelty of the study however, a previously published network meta-analysis entitled, “Hayashi M, Yamamoto N, Kuroda N, Kano K, Miura T, Kamimura Y, Shiroshita A. Peripheral Nerve Blocks in the Preoperative Management of Hip Fractures: A Systematic Review and Network Meta-Analysis. Ann Emerg Med. 2024 Jun;83(6):522-538. doi: 10.1016/j.annemergmed.2024.01.024. Epub 2024 Feb 22. PMID: 38385910” highlighted a similar subject. The author should be describing more reasons regarding the uniqueness of the study.

**Answer:** Thanks for your comments and valuable advice. Thank you for your comments on the paper, which are very helpful in improving the quality of our manuscript. We have revised our paper according to your insightful advice.

**Specific Comments:**

1. Introduction: Lines 47-48: The author states the possible prevalence projection of hip fracture; however, the author needs to add statistical evidence from the listed references to provide support for their statement.

**Answer:** Thank you for the comments on the paper. We have revised the manuscript based on the reviewers’ suggestions. (Line 48-51).

2. Lines 48-50: the author cited the 2011 Association of Anaesthetists, the author should cite the recent guideline published in 2020.

**Answer:** Thank you for your kind comments. We have updated the reference. (Line 54, 259-261).

3. Line 68-69: the author should be emphasizing the hypothesis and not the methodology in the introduction. The statement for the Bayesian NMA and indirect comparison is more suitable in the method section and is irrelevant to the introduction.

**Answer:** Thank you for your pertinent comments, which are of great help in improving the quality of our manuscript. We have removed inappropriate statements from the introduction section. (Line 74-77).

4. Line 52-54: the statement is unclear please rewrite the statement for more clarity.

**Answer:** Thank you for your comments, which are very important to improve the readability of the article. We have revised the manuscript according to the comments of reviewer. (Line 55-58).

5. Line 72-73: the author presents their hypothesis in the former sentence, however, in lines 72-73, the author should state that they will assess the duration of lower limb weakness as a secondary outcome, however, the written statement looks like the author is quite sure about the possible outcome and its risk regarding the weakness. This statement is not relevant in writing an introduction.

**Answer:** Thank you for your comments and advice. We apologize for any confusion caused by the unclear expression in our manuscript. In fact, our hypothesis encompasses both the analgesic efficacy and safety of FICB, and we have integrated these two aspects. Furthermore, in our study, we will evaluate the duration of lower limb weakness as a secondary outcome. (Line 74-79, 123, 178, 179).

6. Methods:

Line 91-92: the author included only the Fascia iliaca block as an intervention in the search strategy. Similarly, table S1 also shows the strategy only restricted to the following keywords. In contrast, the network diagram shows systemic analgesics, similarly, the inclusion criteria also include systemic analgesics in lines 107-108. Therefore, systemic analgesia should also be included in the search strategy to provide the rank-based analysis of all the interventions considering the author is planning to conduct a network meta-analysis. However, according to the hypothesis, the author seems only interested in the single and continuous FICB so a meta-analysis or a network analysis only with the three interventions: single, continuous FICB and placebo should be conducted, along with an update of the illustrated network plot will be required. The author needs to refine and redefine their methodology, search strategy, PICO and hypothesis regarding the inclusion or exclusion of systemic analgesics.

**Answer:** Thank you for your valuable suggestions and reminders, which are of great help to improve the logic and readability of the article. We carefully checked the inclusion and exclusion criteria of our manuscript, revised the contradictions, and updated table S2 and Fig 2. (Line101, 102, 117-119, also see table S2 and Fig 2).

7. Line 164: the statement is unclear.

**Answer:** Thank you for your comments. We have removed the ambiguous expression. (Line 209).

8. Discussion

Line 181: the author claims the novelty of the study however, a previously published network meta-analysis entitled, “Hayashi M, Yamamoto N, Kuroda N, Kano K, Miura T, Kamimura Y, Shiroshita A. Peripheral Nerve Blocks in the Preoperative Management of Hip Fractures: A Systematic Review and Network Meta-Analysis. Ann Emerg Med. 2024 Jun;83(6):522-538. doi: 10.1016/j.annemergmed.2024.01.024. Epub 2024 Feb 22. PMID: ” highlighted a similar subject. The author should be describing more reasons regarding the uniqueness of the study.

**Answer:** Thank you for your suggestions and reminders, which are of great help in improving readers' understanding of our articles. We have described the differences between our research and published systematic reviews, and the uniqueness of our systematic reviews. (Line 220-229).
