# Supplementary material for: Comparison of traditional systemic analgesic, single shot or continuous fascia iliaca compartment block for pain management in patients with hip or proximal femoral fractures: A protocol for systematic review and network meta-analysis
Source: PLoS One. 2025 Mar 26;20(3):e0319988. doi: 10.1371/journal.pone.0319988 (PMC11940658; doi:10.1371/journal.pone.0319988)
Supplement: Attachment — Submitted filename: Response_to_Reviewers_auresp_3.docx [file pone.0319988.s007.docx]

Jiaxi Tang MD. PhD

**Department of Anesthesiology, Chongqing Key Laboratory of Translational Research for Cancer Metastasis and Individualized Treatment, Chongqing University Cancer Hospital,**

Chongqing 400030, China

E-mail: tangjiaxi1029@126.com

February 5, 2025

**RE:  Manuscript ID:** PONE-D-23-33546R2

Dear Editor，

We would like to thank the editor for giving us a chance to resubmit the paper, and also thank the reviewer for giving us constructive suggestions which would help us in depth to improve the quality of the paper. Here we submit a new version of our manuscript with the title “**Comparison of traditional systemic analgesic, Single shot or continuous Fascia Iliaca Compartment Block for pain management in patients with hip or Proximal Femoral Fractures: A protocol for systematic review and network meta-analysis**”, which has been modified according to the reviewer and editor’s suggestions. We highlight all the changes using a track change function in the revised manuscript.

Sincerely yours,

Jiaxi Tang MD. PhD

-------------------------------------------------------------------------------------------------------

The following is a point-by-point response to the editor and reviewer’s comments.

**Editor:**

**Specific comments:**

1. As the Jadad score is only used for inclusion decisions and not for quality appraisal, it should be deleted from the abstract

**Answer:** Thank you for your valuable feedback. We understand that the Jadad score is primarily used for the inclusion decision rather than as a comprehensive quality appraisal tool. To avoid any confusion, we will revise the manuscript according to your suggestion. (Line 39)

2. Please make sure that in the abstract and in the text, you refer to the appraisal tool as ROB 2 to avoid misunderstandings

**Answer:** Thank you for your insightful suggestion. We agree that referring to the appraisal tool as ROB 2 is more appropriate to avoid any confusion with other quality assessment tools. We will revise both the abstract and the main text of the manuscript to consistently refer to the tool as ROB 2, ensuring clarity for the readers. (Line 39,163)

3. Please mention the use of the GRADE approach in the abstract. In the text, please make sure that you use an appropriate original reference for GRADE and describe the instrument and the process in some more detail. For example, are you planning to use GRADEpro? Please mind that results of the GRADE assessments are “certainty (not grade) of the evidence”

**Answer:** Thank you for your helpful suggestion. We will revise the abstract to include a mention of the GRADE approach and its role in assessing the certainty of the evidence. In the main text, we will ensure that we use the appropriate original reference for GRADE and provide a more detailed description of the instrument and the process. Specifically, we plan to use GRADEpro to conduct the GRADE assessments, and we will clarify that the results of the GRADE evaluation refer to the certainty of the evidence. We will also make sure to use the correct terminology and methodology to avoid any confusion. (Line 39,40,168-174，445-449)

4. For screening of results, EndNote seems not sufficient, please state the use of an appropriate software as e.g. Covidence, Rayyan or EppiReviewer

**Answer:** Thank you for your suggestion. We agree that using specialized software for screening, such as Covidence, Rayyan, or EppiReviewer, would be more appropriate. We will revise the manuscript to reflect the use of EndNote and Rayyan for the screening process to improve the efficiency and accuracy of the review. (Line 119-123)

**Reviewer #3:**

**General comments:**

1. Does the manuscript provide a valid rationale for the proposed study, with clearly identified and justified research questions?

The research question outlined is expected to address a valid academic problem or topic and contribute to the base of knowledge in the field.

Reviewer #3: Yes

________________________________________

2. Is the protocol technically sound and planned in a manner that will lead to a meaningful outcome and allow testing the stated hypotheses?

The manuscript should describe the methods in sufficient detail to prevent undisclosed flexibility in the experimental procedure or analysis pipeline, including sufficient outcome-neutral conditions (e.g. necessary controls, absence of floor or ceiling effects) to test the proposed hypotheses and a statistical power analysis where applicable. As there may be aspects of the methodology and analysis which can only be refined once the work is undertaken, authors should outline potential assumptions and explicitly describe what aspects of the proposed analyses, if any, are exploratory.

Reviewer #3: Yes

________________________________________

3. Is the methodology feasible and described in sufficient detail to allow the work to be replicable?

Descriptions of methods and materials in the protocol should be reported in sufficient detail for another researcher to reproduce all experiments and analyses. The protocol should describe the appropriate controls, sample size calculations, and replication needed to ensure that the data are robust and reproducible.

Reviewer #3: Yes

________________________________________

4. Have the authors described where all data underlying the findings will be made available when the study is complete?

The PLOS Data policy requires authors to make all data underlying the findings described in their manuscript fully available without restriction, with rare exception, at the time of publication. The data should be provided as part of the manuscript or its supporting information, or deposited to a public repository. For example, in addition to summary statistics, the data points behind means, medians and variance measures should be available. If there are restrictions on publicly sharing data—e.g. participant privacy or use of data from a third party—those must be specified.

Reviewer #3: Yes

________________________________________

5. Is the manuscript presented in an intelligible fashion and written in standard English?

PLOS ONE does not copyedit accepted manuscripts, so the language in submitted articles must be clear, correct, and unambiguous. Any typographical or grammatical errors should be corrected at revision, so please note any specific errors here.

Reviewer #3: Yes

**Answer:** Thank you for your comments and recognition of our research.

**Specific Comments:**

1. Subgroup Analysis

Consider including a sensitivity analysis to assess the robustness of findings.

**Answer:** Thank you for your suggestion. We appreciate the importance of sensitivity analysis in evaluating the robustness of our findings. We would like to clarify that sensitivity analysis is already included in our methodology, specifically by changing the pooled model, conducting meta-regression, and excluding low-quality studies. To enhance clarity, we have now explicitly separated sensitivity analysis from subgroup analysis in the text and provided further details to emphasize its role in confirming the robustness of our conclusions. (Line 249-251, 260-270)

2. Discussion

More emphasis should be placed on the practical implications of using FICB over systemic analgesia, particularly in resource-limited settings.

**Answer:** Thank you for your valuable feedback. We agree that emphasizing the practical implications of using fascia iliaca compartment block (FICB) over systemic analgesia, especially in resource-limited settings, is crucial. We have revised the discussion section to further elaborate on how FICB can be a more effective and feasible option in such environments. Specifically, we have highlighted its advantages in reducing opioid consumption, minimizing systemic side effects, and potentially improving patient outcomes in settings where access to multimodal analgesia and advanced monitoring may be limited. We appreciate your insightful suggestion and believe this revision strengthens the clinical relevance of our study. (Line 311-321).
